# Supplementary material for: Activation of Mitochondrial 2-Oxoglutarate Dehydrogenase by Cocarboxylase in Human Lung Adenocarcinoma Cells A549 Is p53/p21-Dependent and Impairs Cellular Redox State, Mimicking the Cisplatin Action
Source: Int J Mol Sci. 2020 May 26;21(11):3759. doi: 10.3390/ijms21113759 (PMC7312097; doi:10.3390/ijms21113759)
Supplement: Supplementary file 1 [file ijms-21-03759-s001.pdf]

Activation of Mitochondrial 2-Oxoglutarate Dehydrogenase by Cocarboxylase in Human Lung Adenocarcinoma Cells A549 is p53/p21-Dependent and Impairs Cellular Redox State, Mimicking the Cisplatin Action

Victoria I. Bunik, Vasily A. Aleshin, Xiaoshan Zhou, Vyacheslav Yu. Tabakov and Anna Karlsson

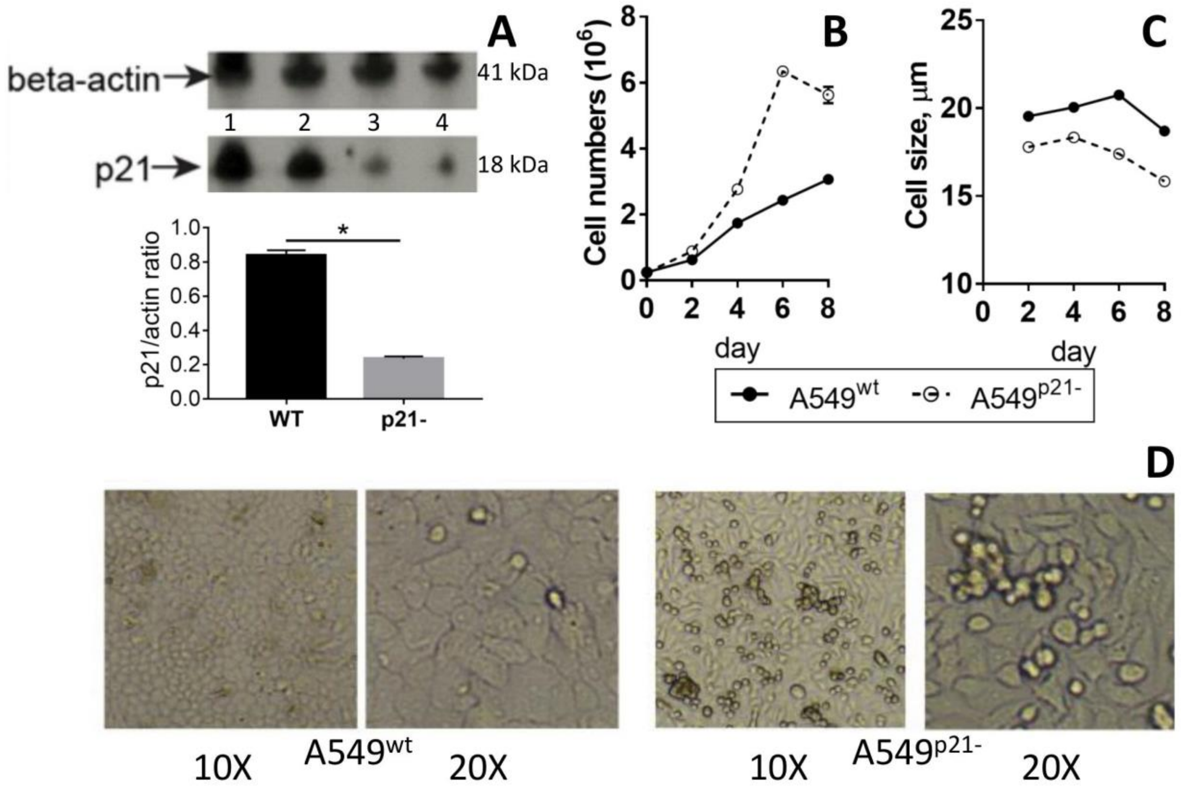

Figure S1. Comparison of A549^{p21-} and A549^{wt} cells. 25000 cells were seeded in 6-well plates and cultured in standard DMEM. **A**. The levels of p21 and beta-actin proteins, detected by Western blotting in A549^{p21-} cells (lanes 3, 4), compared to A549^{wt} cells (lanes 1, 2). Quantification was done using ImageJ2 (https://imagej.net/ImageJ2). The data are presented as mean ± SEM (*, $p<0.05$ by unpaired *t* test with Welch's correction). **B**. Time-dependence of the cell number. **C**. Time-dependence of the cell diameter **D**. Images of the cell morphology at 10x and 20x zooming.

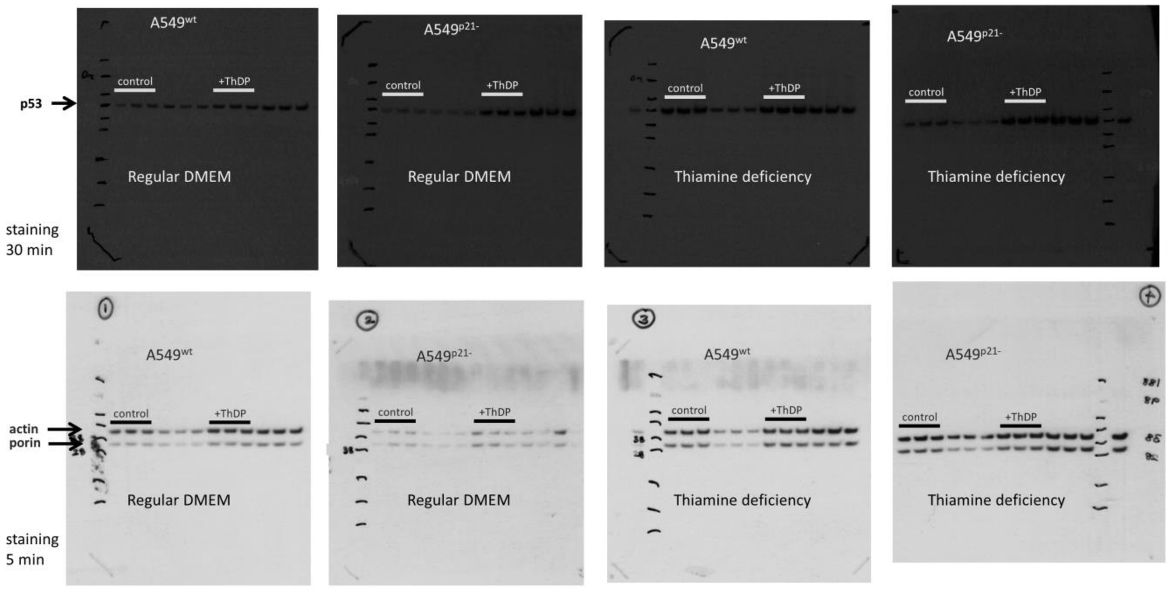

Figure S2. Uncropped western blots on the p53 levels (upper blots) and normalization to actin and VDAC/porin (bottom blots) under varied ThDP saturation in A549^{wt} (1st and 3rd columns) and A549^{p21-} (2nd and 4th columns) cells. Conditions of the cell cultures are as indicated on the blot pictures: the regular DMEM (1sr and 2nd columns) or the thiamine deficient DMEM (3rd and 4th columns). +ThDP – addition of 5 mM ThDP.
